# Making precision breeding bloom: APL is a promising target to engineer flowering time

**DOI:** 10.1093/plcell/koag299

**Published:** 2026-09-30

**Authors:** Vanessica Jawahir

**Affiliations:** Assistant Features Editor, The Plant Cell, American Society of Plant Biologists; Donald Danforth Plant Science Center, Saint Louis, MO, United States

Building a perfect plant that possesses all the desirable traits in a specific environment is the core of plant breeding. Precision breeding involves using gene editing, with tools such as CRISPR/Cas9, base-editing, or other site-directed nucleases to precisely modify specific DNA targets to produce a desired effect (Kamburova et al. 2017). Precision breeding touts the ability to speed up the development of new cultivars and make improvements in species with low genetic diversity and long-lived species, such as trees (Sheridan 2025). With the invention and improvement of gene editing technologies, regulatory approval of their use, and identification of gene targets for specific trait improvement, we are one step closer to the ability to pick and choose which traits we want a plant to have.

In new work, **Zhiwei Zhao and colleagues** (Zhao et al. 2026) identified a protein domain that can be targeted for precision breeding to fine-tune flowering time across species. This discovery started by characterizing EMS-induced *additive delayed-flowering* mutant (*edf1*) in maize. *edf1* flowered later than wild-type in both long- and short-day photoperiods in the field, while heterozygous *edf1* flowered between wild-type and the homozygous mutant. Homozygous *edf1* plants were taller and had greater fresh weight biomass compared to wild-type, a desirable trait in silage maize (Fig. 1).

**Figure 1:**
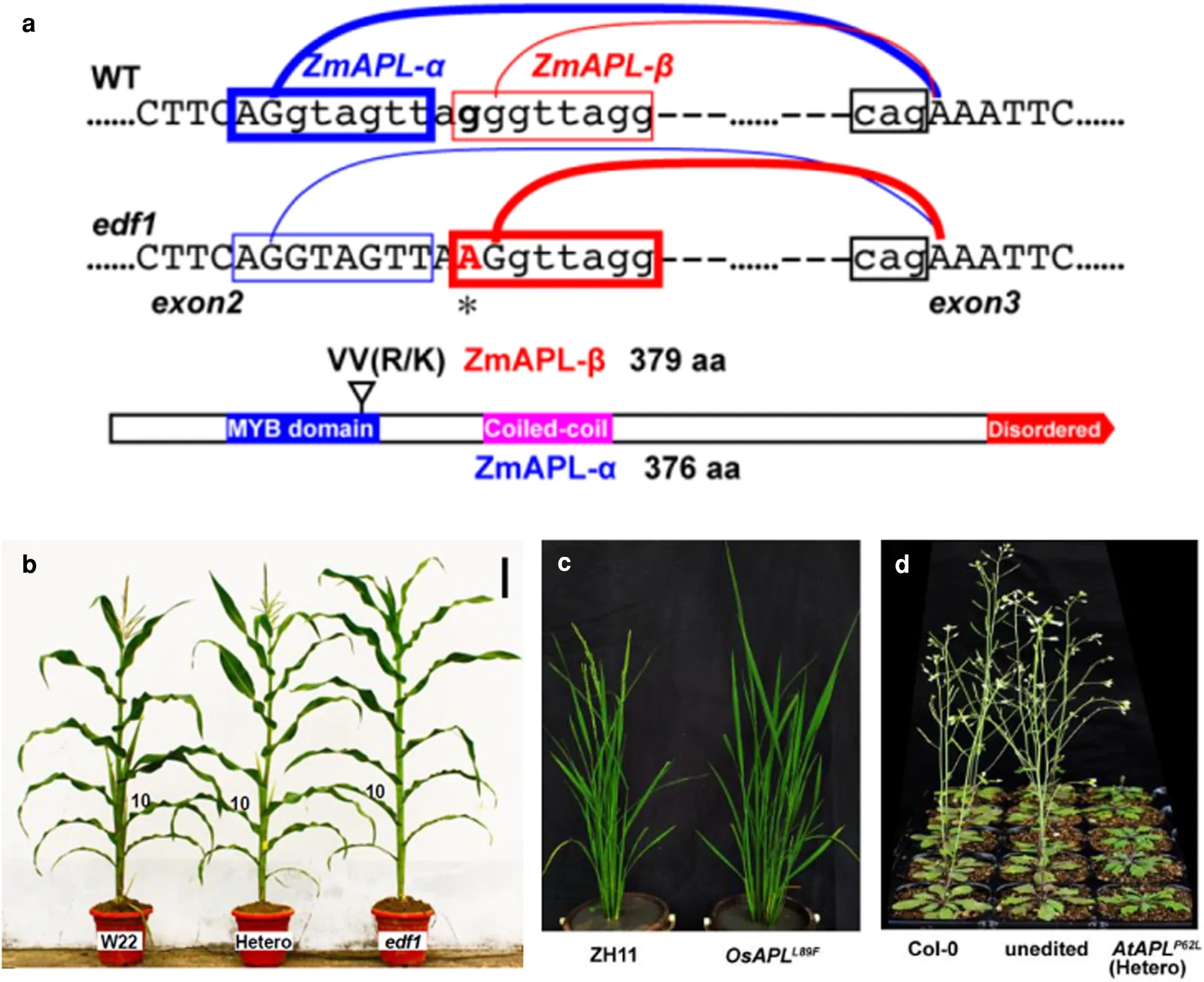
a) The causative SNP of *edf1* alters abundance of *ZmAPL* isoforms, leading to increased expression of a protein with an extra 3 amino acids in the MYB domain. Mutations in the MYB domain of APL delay flowering time in maize b), rice c), and Arabidopsis d). In all panels, the wild-type plant is on the left and the mutant is on the right. Figure adapted from Zhao et al. (2026), Figs. 1, 2, and 6.

The causative mutation in *edf1* was identified as a point mutation in *Zm00001eb233310 (ZmAPL),* homologous to the MYB transcription factor *ALTERED PHLOEM DEVELOPMENT (APD)* in Arabidopsis. In wild-type plants, *ZmAPL* produces 3 splice variants designated *ZmAPL-α*, *ZmAPL-β*, and *ZmAPL-γ*, with *ZmAPL-α* being the dominant variant. The causative single nucleotide polymorphism (SNP) in *edf1* changes a weakly conserved native 5′ splice-donor site to a canonical and stronger splice-site signal in the MYB domain which increases the amount of *ZmAPL-β* isoform (Fig. 1). The protein encoded by *ZmAPL-β* is 3 amino acids longer than that encoded by *ZmAPL-α*. Transgenic complementation of *edf1* with *ZmAPL-α* partially rescued the delayed-flowering phenotype while RNAi knockdown lines recapitulated the delayed-flowering phenotype, confirming that the mutation in *ZmAPL* is responsible for the flowering phenotype of *edf1*.

In Arabidopsis, *APL* is essential for vasculature system development and is also a regulator of flowering time (Bonke et al. 2003; Abe et al. 2015). Arabidopsis *APL* knockout mutants are seedling-lethal while point mutations delay flowering time in long-day conditions. In this new work, Zhao et al. similarly found that their CRISPR-Cas9 knockout mutant of *ZmAPL* is seedling-lethal and displayed aberrant vasculature morphology. The authors sought to understand how the altered *ZmAPL* isoform abundance in *edf1* leads to a delayed flowering phenotype without severe vasculature defects. RNA sequencing on their CRISPR knockout mutant *apl,* the *edf1* splicing defect allele, and wild-type controls revealed that differentially expressed genes in *apl* were enriched for chromatin and nucleosome processes, which explains the pleotropic and severe phenotypes of *APL* knockout mutants. Contrastingly, *edf1* was enriched for pathways linked to flowering time and biomass, such as genes involved in circadian rhythms and photosynthesis. Specifically, the *ZEA CENTRORADIALIS (ZCN)* family genes *ZCN7*, *ZCN8*, and *ZCN12*, which are known flowering promotion genes, were downregulated in *edf1* suggesting that *ZmAPL-α* promotes flowering by regulating the *ZCN* gene family (Meng et al. 2011). *ZCN8*, the primary flowering activator, rapidly increases expression around the floral-transition phase in wild-type while expression remains low in *edf1*, supporting that the model that *edf1* mutation disrupts flowering via *ZCN8*.

The authors next investigated whether ZmAPL directly regulates ZCN8 expression, *ZmAPL-α* but not *ZmAPL-β* activated expression of luciferase under control of the *ZCN8* promoter in a transient dual-luciferase system. Expressing both *ZmAPL-α* and *ZmAPL-β* produced intermediate levels of luciferase, indicating that *ZmAPL-β* antagonizes the activity of *ZmAPL-α*. A direct interaction of ZmAPL with the *ZCN8* promoter was corroborated with chromatin-immunoprecipitation (ChIP)-qPCR, which found enrichment of ZmAPL-α at *ZCN8* promoter fragments in wild-type with no enrichment in the *edf1* mutant, supporting that the ZmAPL-β isoform cannot associate with the *ZCN8* promoter.

APL forms both homo- and heterodimers and these protein-protein interactions are necessary for binding DNA. Yeast two-hybrid assays and bimolecular fluorescence complementation assays found that ZmAPL-α forms a stable homodimer with itself and can form a heterodimer with ZmAPL-β. Modeling with AlphaFold predicted that the ZmAPL-α/ZmAPL-α is stable homodimer while the ZmAPL-α/ZmAPL-β heterodimer fails to stably bind DNA. These experiments suggest a model in which ZmAPL-α activates expression of *ZCN8* to promote flowering, but the presence of excessive ZmAPL-β such as caused by the *edf1* mutation may lead to more ZmAPL-α/ZmAPL-β heterodimer formation, therefore reducing expression of *ZCN8* and resulting in delayed flowering.

The authors reasoned that *APL* is a prime target for precision breeding to control flowering time because various alleles exhibit a range of flowering phenotypes and APL proteins are highly conserved across plant species. Base-editing technology was used to target the MYB domain of APL homologs in rice and Arabidopsis and successfully produced delayed flowering phenotypes in both species demonstrating this is a viable genetic target across plant species.

In summary, Zhao et al. (2026) elegantly demonstrated that targeted edits to the MYB domain of APL can be used to fine-tune flowering time in both monocots and dicots while avoiding the seedling lethality caused by complete APL loss (see Fig. 1). *APL* could be a prime candidate for precision breeding in species and lines where an increase in biomass is desired, such as silage maize, or increasing plant success when introduced to a new environment with a different photoperiod. Zhao et al. (2026) discovered a useful gene target to control flowering timing from a forward genetic screen by identifying a splice-site mutation in *APL* which alters the protein's binding affinity for the promoter of the flowering regulator *ZCN8*. This study has broad implications that changes to the MYB domain of APL have cross species functionality and relevance as a target for precision breeding.

## Recent related articles in *The Plant Cell*

- Zhang et al. (2026) describe that EAR motif-containing protein (ECAP) interacts with FLOWERING CONTROL LOCUS A (FCA) to mediate repression of *FLOWERING LOCUS C* (*FLC*) in *Arabidopsis*.
- Zhou et al. (2026) describe how the *Arabidopsis* Splicing factor 3B subunit 2 (SF3B2) negatively regulates floral transition by modulating splicing of its target genes which include *FLOWERING LOCUS C* (*FLC*).
- Wu et al. (2025) demonstrate that the blue light receptor *FLAVIN-BINDING KELCH REPEAT F-BOX 1a* (ZmFKF1a) is controlled by the evening complex which fine-tune photoperiodic flowering time in maize independently of the *ZCNs*.

## Data Availability

No new data were generated in the preparation of this manuscript.
